# Multicentre cross-sectional clinical evaluation study about quality of life in adults with disorders/differences of sex development (DSD) compared to country specific reference populations (dsd-LIFE)

**DOI:** 10.1186/s12955-018-0881-3

**Published:** 2018-04-03

**Authors:** Marion Rapp, Esther Mueller-Godeffroy, Peter Lee, Robert Roehle, Baudewijntje P. C. Kreukels, Birgit Köhler, Anna Nordenström, Claire Bouvattier, Ute Thyen, on behalf of the dsd-LIFE group, Birgit Kohler, Peggy Cohen-Kettenis, Annelou de Vries, Wiebke Arlt, Claudia Wiesemann, Jolanta Slowikowska-Hilczer, Aude Brac de la Perriere, Charles Sultan, Francoise Paris, Claire Bouvattier, Ute Thyen, Nicole Reisch, Annette Richter-Unruh, Hedi Claahsen-van der Grinten, Anna Nordenstrom, Catherine Pienkowski, Maria Szarras-Czapnik

**Affiliations:** 1 Klinik fur Kinder- und Jugendmedizin, Universitat zu Lubeck, Ratzeburger Allee 160, 23538 Lubeck, Germany; 2 Pediatrics, Penn State College of Medicine, Hershey, PA 17033 USA; 3 Charite, Campus Virchow-Klinikum, Koordinierungszentrum fur Klinische Studien (KKS Charite), 13353 Berlin, Germany; 4 Medische Psychologie en Medisch Maatschappelijk Werk, VU Medisch Centrum, PO Box 7057, 1007 MB Amsterdam, The Netherlands; 5 Charite, Campus Virchow-Klinikum, Klinik fur Padiatrie mit Schwerpunkt Endokrinologie und Diabetologie, 13353 Berlin, Germany; 6 Department of Women’s and Children’s Health, Karolinska Institutet, Karolinska University Hospital, 171 76 Stockholm, Sweden; 7 Endocrinologie pediatrique, Centre de reference des maladies rares du developpement sexuel, Hopital Bicetre, Universite Paris-Sud, 78 rue du General Leclerc, 94270 Le Kremlin Bicetre, France

**Keywords:** Disorders of sex development, Quality of life, Rare disorders, Health status, General population

## Abstract

**Background:**

Previous studies in quality of life (QOL) in individuals with disorders/differences of sex development (DSD) have been restricted to subpopulations of the condition. We describe QOL in adult persons with DSD compared to country specific references and assess the impact of diagnosis.

**Methods:**

The multicentre cross-sectional clinical evaluation (dsd-LIFE) took place in 14 specialized clinics in six European countries. Adolescents (≥16 years) and adults having a DSD condition were included from 02/2014 to 09/2015. The main outcome QOL was measured by the WHOQOL-BREF (domains of *physical health*, *psychological*, *social relationships*, and *environment*). QOL was compared to country specific reference populations by using unpaired t-tests. Linear regression models explained the additional variance of the diagnosis on QOL.

**Results:**

Three hundred one individuals with Turner Syndrome, 219 with Klinefelter Syndrome (including XYY), 226 with 46,XX CAH and 294 with rare DSD conditions (gonadal dysgenesis, androgen insensitivity syndrome, severe hypospadias, and androgen synthesis errors or other diagnosis) took part. Compared to healthy European populations, QOL was similar in *psychological*, slightly worse in *physical health*, and slightly better in *environment*. In *social relationships,* QOL was significantly poorer compared to healthy and non-healthy reference populations. In linear regression models health status was the most important predictor of QOL; additional variance was explained by feelings about household’s income in all domains, and the relationship status in *social relationships*. Diagnosis explained nearly no additional variance.

**Conclusions:**

Except for social relationships, most people with DSD adapt well to their life circumstances and report a good QOL. Not diagnosis, but the individual’s health status is much more important than previously thought. Therefore care for people with DSD should focus more on chronic physical or mental health problems both related and unrelated to the diagnosis itself.

**Trial registration:**

German Clinical Trials Register DRKS00006072.

## Background

Disorders of sex development (DSD) are defined as congenital conditions in which the development of chromosomal, gonadal or anatomic sex is atypical, following the statement of the Chicago Consensus Meeting in 2005 [1]. DSD contains sex chromosome conditions (including Turner Syndrome (TS), Klinefelter syndrome (KS) and mixed gonadal dysgenesis (GD), conditions with a 46,XY karyotype (including complete/partial androgen insensitivity syndrome (AIS), complete/partial GD, steroid synthesis errors and severe hypospadias) and conditions with a 46,XX karyotype (including Congenital Adrenal Hyperplasia (CAH), GD and XX men). After the consensus meeting many debates about the acronym DSD and the term disorders of sex development followed. We agreed in dsd-LIFE to use the acronym DSD, standing for disorders/differences of sex development, and to refer to the specific condition as often as possible. In this paper we will also use the terms females (women), males (men) and other gender (others) when referring to sex assignment or gender role. Management of DSD conditions is complex, because people with DSD often have other health problems and chronic physical and mental diseases both related and unrelated to the specific DSD diagnosis. To emphasize this aspect an update of the recommendation of the Chicago Consensus Meeting was released in 2016, which stated that one major aim in the treatment of people with DSD should be to reach the best possible quality of life (QOL) for everybody [2].

The World Health Organisation defines QOL as ‘an individual’s perception of their position in life in context of the culture and value systems in which they live, and in relation to their goals, expectations, standards and concerns [3]. During the 1990’s, the WHOQOL assessment group initiated a project to develop a generic instrument for the assessment of QOL worldwide in healthy and non-healthy persons. The WHOQOL–100 was developed using person-centered methods. As the questionnaire was too wide/extensive for epidemiological or cross-sectional studies as well as clinical purposes, an extracted short form, the WHOQOL-BREF, was developed [4, 5]. The instrument covers physical and emotional quality of life, social relations (including social support from friends and satisfaction with sex life) and environment (including satisfaction with physical environment, health care, and information); it does not include measures of role functioning related to physical or mental health issues. Through the last decade QOL was conceptualized to be a measure of the successful adaptation of the individual to his/her condition in life, including to one person’s physical and mental health or to other life events that might influence health in a holistic perspective.

QOL was rarely measured as an outcome in adults with DSD in the past. A review about earlier studies of different XY-DSD conditions did not focus on quality of life, but on functioning or psychological wellbeing [6]. More recent studies about patient-reported QOL in adult people with DSD often lack sufficient sample sizes [6–8], include a mixture of divers diagnoses [9], focus on the more common conditions in 46,XX- or 46,XY-DSD [6, 10, 11], or are hampered by large selection biases [12, 13]. Recently two studies were published that included people with 46,XY DSD living as males; [14, 15] only one included people not identifying as female or male gender as well [15]. Published QOL studies in people with DSD reported inconsistent results for those with sex chromosome conditions [8, 16, 17], and for conditions with a 46,XX or 46,XY karyotype, both in qualitative or quantitative studies [11]. Compared to reference populations, some studies reported worse QOL [8, 17], others similar [9, 15, 16] or even a better QOL of people with DSD than the controls [10–12]. Quite recently the WHOQOL-BREF was first used to assess the QOL in people with 46,XX and 46,XY karyotype DSD conditions in Brazil, China and Italy [11–13].

The cross-sectional clinical evaluation study dsd-LIFE is therefore the first large multi-centre European study of people with DSD [18]. Dsd-Life evaluates whether clinical treatment is effective from a subjective point of view, has a reasonable risk-benefit balance, and improves quality of life.

The objectives of this report are to examine, whether:

1. QOL in individuals with DSD differ from the general (reference) population.
2. the specific diagnoses explains additional variance of QOL after taking gender, age, socioeconomic and maritual status, and the overall health status into account.

## Methods

### Study design

The methods of the multicentre cross-sectional clinical evaluation study dsd-LIFE, described in detail elsewhere [18], are summarised below. The dsd-LIFE consortium consisted of 16 European partners from Germany, France, the Netherlands, Poland, Sweden and the United Kingdom (UK), of whom fourteen were active recruiting sites. Recruitment of adolescents age 16 onwards and adults with DSD through patients’ advocacy groups and clinical records took place from February 1st, 2014 to September 30th, 2015. A total number of 3100 eligible people were approached, of whom 1040 took part in the study. All those met the inclusion criteria for having a DSD condition as described in the classification system of the Chicago Consensus Conference [1].

Dsd-LIFE consisted of two study parts. The first part included a medical interview, a retrospective chart review and medical examinations; all carried out by trained researchers following standard operation procedures. The second part of the study consisted of the patient reported outcome (PRO) questionnaire. The PRO was administered as an online version, accessible only with a secure password on the recruitment centres; if needed, a paper-pencil version was provided as well. The PRO included sociodemographic data (including age, the ESISCED as an European standardised education measurement, feeling about household income, relationship status with having a partner, being single or living with parents, and information about the participants health status with the general question ‘How is your health in general? Would you say it is: (very) bad to (very) good.’), and standardized questionnaires about the general quality of life (e.g. WHOQOL-BREF), psychological well-being, psychosexual development, sexuality and condition specific self-constructed items.

### Outcome measurement – Quality of life

The instrument WHOQOL-BREF comprises 24 items, resulting in four domains (physical health, psychological, social relationships, and environment) with three to eight items per domain [4]. It is validated for people, aged 18 years and older [4]. All answers are presented with a five-point-Likert scale. Higher scores indicate a higher quality of life. Domains are not scored when two or more items are missing (or 1-item in the 3-item domain social relationship) and then transformed on a scale from 0 to 100 or from 4 to 20 in same studies to enable comparisons between domains. The WHOQOL-BREF has no global score. The domains show good psychometric properties without ceiling or floor effects and an internal consistency of Cronbach’s alpha being ≥0.8 for every domain, except for social relationships with 0.68 [5]. The WHOQOL-BREF was developed for cross-cultural comparisons of QOL and is available in more than 40 languages, including all dsd-LIFE languages [4]. Used in equal or similar cultural contexts like in-between Europe, national weightings are not needed in analyses [19].

### Reference data: Comparison data for each participating country

#### France

Dsd-LIFE participants from France were compared to an adult sample of 16,450 randomly selected people, 18–75 years old, drawn from the National Health Barometer 2005, a periodic study by the French National Institute for Preventive and Health Education [20]. The study population included 6808 male and 9584 female participants. 1447 were young adults (18 to 24) and 2313 elderly people (65–75 years old). Self-reported chronic physical or mental disease was described in 4192 participants. For economical reasons the environment domain was not assessed in the National Health Barometer. The survey used a computer-assisted telephone interview system.

#### Germany

Dsd-LIFE participants from Germany were compared to a representative urban sample of 2073 adults (≥18 years) of the general population [21]. For 2055 participants data was included in the analysis: 927 males and 1128 females participated; 240 were young adults (18 to 25 years) and 393 elderly people (≥66 years). Additionally 359 patients with physical (*n* = 261) or mental (*n* = 98) chronic diseases were investigated in two university hospitals; one in the Eastern and one in the Western part of Germany. The German reference population was part of the first evaluation study of the WHOQOL-BREF [5].

#### The Netherlands

Dsd-LIFE participants from the Netherlands were compared to a sample of non-healthy persons with a mental chronic disease and their healthy control group, matched for age and sex ratio [22]. 410 psychiatric outpatients of one community mental health centre completed the WHOQOL-BREF during the study period from March 2001 to March 2002. No persons with severe mental illness or mental retardation and some further exclusion criteria took part. All participants were of Dutch ethnic origin and between 21 to 50 years of age. 41% of the participants were male with a mean age of 34.8 years, 59% female with a mean age of 32.5 years. The matched reference group was taken from a pooled data set based on Dutch general population studies (1999–2002).

#### Poland

Dsd-LIFE participants from Poland were compared to a Polish study about the QOL in 438 healthy and 470 non-healthy people [23]. The age ranged from 18 to 85 years, mean age of the healthy people was 25 years (18–59 years) and for the unhealthy 44 years (18–85 years). Gender differed between both groups with 60% females in the healthy people and 69% females in the non-healthy. The non-healthy people suffered various physical and mental diseases and were recruited from five in-patient wards and four out-patient clinics. The healthy people were a little more educated (often students or health care professionals) and more often living as single than the non-healthy people. Both samples were recruited in the same Polish region.

#### Sweden

No data from the general population or other validation data were available for comparison.

#### UK

Dsd-LIFE participants from the UK were compared to an assessment about the psychometric properties and results of the WHOQOL-BREF in healthy and non-healthy people all over the UK. The sample included convicted individuals in prisons and a sample undergoing plastic or lifestyle surgery which was excluded from our reference group [24]. We compared our data to 1328 healthy people and 1864 people with various physical and mental disorders, treated at multiple settings all over UK. The healthy people included students and student nurses. Age range for the whole cohort was 16–105 years with a mean age of 45 years; overall 64% females took part in the study.

### Statistics

Analyses were done separately for each of the four WHOQOL-BREF domains. We first examined the psychometric properties of the self-reported WHOQOL-BREF scores in adults with DSD. We assessed the differences between the QOL of our study participants with the QOL of published country specific reference cohorts using unpaired *t*-tests. For adjusting significance levels to account for multiple comparisons we used Bonferroni corrections.

For the following analyses we grouped participants into five categories: female TS, male KS (including male XYY), female CAH, female XY-DSD and male XY-DSD. Male and female XY-DSD groups comprised the diagnoses: (complete/partial/mixed) GD with all karyotype differences, complete/partial AIS, androgen synthesis defects, severe hypospadias and other rare diagnosis not included into female TS, male KS and female CAH. Participants who identified other than male or female gender or not the typical gender for the condition are excluded from the diagnosis specific analyses (*n* = 18). This was necessary due to a high co-linearity between diagnosis group and gender.

We compared the QOL per diagnosis group by using analysis of variance and assessed the covariate- adjusted effect of the diagnosis on QOL by multiple linear regression. The regression models included age, feelings about household’s income as a surrogate for economical status, marital status and the overall health status. Adjustment for educational level was not necessary as there was no association in univariate analyses. Participants with partly missing data were excluded for the respective domain. Dealing with missing item data followed the procedures as stated in the manual of the WHOQOL-BREF [4]. Feelings about household income were imputed for analysis, but no further imputation techniques were used in the linear regression models. Significance was set at *p* < 0.05. The statistical software statistical analysis system (SAS Version 9.4) and R environment [25] were used for analysis.

## Results

Among the 1040 study participants, 301 had TS (150 with 45,X, 31 with 45,X/46XX, and 120 participants with other TS specific karyotypes), 219 KS (204 with 47,XXY, 6 with 47,XXY/46,XY, eight with other KS specific karyotypes, and one with XYY), 226 CAH (46,XX CAH: 111 salt-wasting, 66 simple virilising, 34 non-classical, and 15 with other CAH specific enzyme errors), and 294 rare conditions (including 45,X/46XY (*n* = 45), 46,XX (*n* = 27) and 46,XY karyotypes (*n* = 222)) [Table 1]. 46,XY karyotypes comprised: 21 complete GD, 37 partial GD, 71 complete AIS, 35 partial AIS, 25 severe hypospadias, and 35 people with androgen synthesis errors or other very rare diagnoses [18]. Altogether 717 females, 311 males and 12 people identifying other than female or male took part in the study. 19.4% had a low educational level (*n* = 202), and 128 (12.3%) found it difficult to live on present income. 383 of 1040 had a partner (36.7%); the others were single (23.7%), lived with their parents (30.9%) or in other life circumstances (3.6%). Regarding participant’s health, 367 participants (35.3%) described it as (very) bad or fair; and 479 participants (46.1%) had any additional longstanding illness apart from their DSD condition.

**Table 1:** Characteristics of the study sample (*n* = 1040)

Variables	Categories	Number	%
Diagnosis	Turner syndrome	301	28.9
	Klinefelter Syndrome	218	21.0
	47,XYY	1	0.0
	46,XX CAH	226	21.7
	46,XX DSD - XX gonadal dysgenesis - XX ovotesticular DSD - XX, males	27	2.6
	46,XY DSD - complete/partial XY gonadal dysgenesis - complete/partial androgen insensitivity syndrome - androgen synthesis defects - severe hypospadias - others	222	21.3
	45,X/46XY	45	4.3
Age	mean (SD)	32.4 (13.6)	
	≤ 19 years	193	18.6
	20–24 years	185	17.8
	25–44 years	458	44.0
	45–64 years	178	17.1
	≥ 65 years	26	2.5
Gender	Female	717	68.9
	Male	311	29.9
	Other than female or male	12	1.2
Education	Low (ESISCED 1–2)	202	19.4
	Middle (ESISCED 3–5)	453	43.6
	High (ESISCED 6–7)	266	25.6
	Other	63	60.6
	Not available	56	5.4
Feeling about household income	Living comfortable on present income	339	32.6
	Coping with present income	393	37.8
	Finding it (very) difficult to live on present income	128	12.3
	Other	16	1.5
	Not available	164	15.8
Relationship status	Being single	246	23.7
	Having a partner	382	36.7
	Living with parents	321	30.9
	Other	37	3.6
	Not available	54	5.2
Health status	How is your health in general? Would you say it is …		
	Very bad	13	1.3
	Bad	72	6.9
	Fair	282	27.1
	Good	494	47.5
	Very good	123	11.8
	Not available	56	5.4
	Do you have any longstanding illness? (apart from your condition)		
	No	461	44.3
	Don’t know or not available	100	9.6
	Yes	479	46.1
	If yes, physical health problem	316	–
	Mental health problem	37	–
	Both (physical and mental)	83	–
	Not available	43	–

For the dsd-LIFE study cohort the WHOQOL-BREF showed acceptable to good psychometric properties with the internal consistency of Cronbach’s alpha being 0.71 for *social relationships* and ≥ 0.8 for every other domain [Table 2].

**Table 2:** Psychometric properties of the WHOQOL-BREF for the whole study sample (*n* = 1040)

WHOQOL-BREF domain	Overall			Cronbach’s α (std.)	Standard error Cronbach’s α	Guttman’s lambda 6
Domain	n	Mean	SD			
	1040					
Physical health						
Range 0–100	984	70.1	18.4	0.82	0.015	0.82
Range 4–20		15.2	2.9			
Psychological						
Range 0–100	988	63.9	18.1	0.83	0.015	0.82
Range 4–20		14.2	2.9			
Social relationships						
Range 0–100	987	62.6	20.9	0.71	0.030	0.64
Range 4–20		14.0	3.3			
Environment						
Range 0–100	986	73.0	14.7	0.80	0.014	0.79
Range 4–20		15.7	2.4			

### Comparison to the reference population

**Fig. 1:**
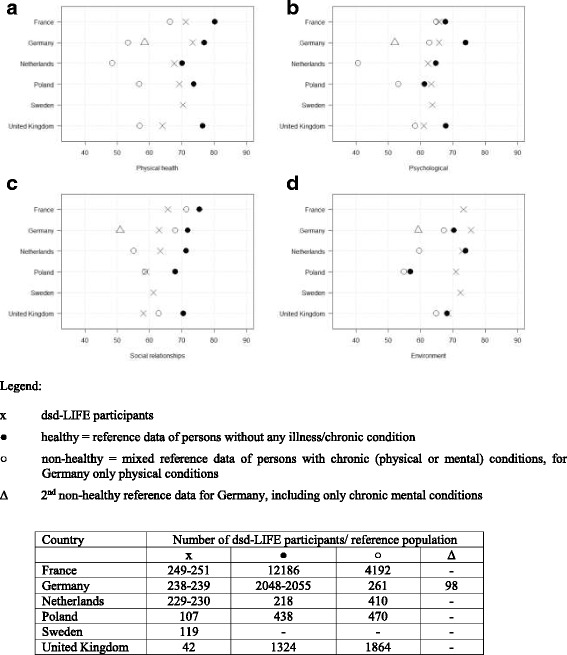
**a**-**d** Comparison of the QOL (WHOQOL-BREF domain) in dsd-LIFE participants with reference populations per country

The QOL of the study participants compared to country specific reference population is shown in [Fig. 1a-d]; the exact scores are presented in Table 3 [Table 3]. The QOL in the domain of *physical health* of persons with DSD was lower than those of the healthy reference population in every country, but higher than those of non-healthy (physical or mental) samples in all countries [Fig. 1a]. Except for France and the UK, the study participants scored closer to the healthy reference population than to the non-healthy samples. In the domain *psychological health* the QOL of the study participants was slightly lower compared to the one of the healthy reference population or even better, like in Poland [Fig. 1b]. In Germany, persons with DSD reported a similar QOL than the non-healthy sample with physical chronic conditions, but study participants scored much higher than the non-healthy sample with mental disorders. Again participants from the UK reported their QOL to be worse and scored close to the non-healthy sample. In the domain of *social relationships* the dsd-LIFE study participants in each country scored much lower than the healthy reference population and in most countries even lower than the non-healthy samples [Fig. 1c]. Regarding the domain *environment* the dsd-LIFE participants of each country reported an equal (Netherlands and UK) or even much better QOL than the healthy reference population (Germany and Poland). For France and Sweden no reference data was available. Overall scores of the study participants were similar for each country per domain, except for the UK whose participants reported lowest QOL in all domains.

**Table 3:** Comparison dsd-LIFE participants to reference populations per country

Country	WHOQOL-BREF	Reference population				Study participants (dsd-LIFE)					*p*-value
			n	Mean	SD	n	Mean	SD	Mean	SD	
							[0–100]	[0–100]	[4-20]	[4-20]	
France											
	Physical health	Healthy	12,186	80.2	11.0	249	71.2	16.8	15.4	2.7	4.1 E-36
	Psychological		12,186	67.7	11.0	251	65.7	17.4	14.5	2.8	4.5 E-03
	Social relationships		12,186	75.4	11.0	250	65.7	19.6	14.5	3.1	1.9 E-41
	Environment		–	–	–	249	73.9	14.6	15.7	2.3	–
	Physical health	Non-healthy	4192	66.4	19.4	249	71.2	16.8	15.4	2.7	1.4 E-04
	Psychological		4192	64.9	13.0	251	65.7	17.4	14.5	2.8	n.s.*
	Social relationships		4192	71.5	19.4	250	65.7	19.6	14.5	3.1	3.9 E-06
	Environment		–	–	–	249	73.3	14.6	15.7	2.3	–
Germany											
	Physical health	Healthy	2052	76.9	17.7	238	73.0	19.3	15.7	3.1	1.3 E-03
	Psychological		2055	74.0	15.7	239	65.0	19.3	14.4	3.1	4.6 E-16
	Social relationships		2048	71.8	18.5	239	62.3	22.2	14.0	3.6	2.9 E-13
	Environment		2053	70.4	14.2	239	75.1	14.6	16.0	2.3	1.6 E-06
	Physical health	Non-healthy (physical)	261	53.4	20.3	238	73.0	19.3	15.7	3.1	2.0 E-25
	Psychological		261	62.7	16.3	239	65.0	19.3	14.4	3.1	n.s.*
	Social relationships		261	68.0	16.9	239	62.3	22.2	14.0	3.6	1.3 E-03
	Environment		261	67.2	13.4	239	75.1	14.6	16.0	2.3	7.4 E-10
	Physical health	Non-healthy (mental)	98	58.5	17.7	238	73.0	19.3	15.7	3.1	5.1 E-10
	Psychological		98	52.0	19.5	239	65.0	19.3	14.4	3.1	4.4 E-08
	Social relationships		98	50.9	26.5	239	62.3	22.2	14.0	3.6	6.3 E-05
	Environment		98	59.3	18.8	239	75.1	14.6	16.0	2.3	3.4 E-15
Netherlands											
	Physical health	Healthy	218	15.2	2.6	229	67.3	20.2	14.8	3.2	n.s.*
	Psychological		218	14.4	2.0	230	61.9	18.1	13.9	2.9	n.s.*
	Social relationships		218	15.4	2.9	230	62.6	21.0	14.0	3.4	3.9 E-06
	Environment		218	15.8	2.0	230	72.6	15.1	15.6	2.4	n.s.*
	Physical health	Non-healthy	410	11.8	3.0	229	67.3	20.2	14.8	3.2	4.7 E-29
	Psychological		410	10.5	2.5	230	61.9	18.1	13.9	2.9	1.4 E-46
	Social relationships		410	12.8	3.5	230	62.6	21.0	14.0	3.4	3.3 E-05
	Environment		410	13.5	2.5	230	72.6	15.1	15.6	2.4	1.3 E-22
Poland											
	Physical health	Healthy	438	15.8	2.2	107	69.1	14.0	15.1	2.2	2.5 E-03
	Psychological		438	13.8	2.5	107	63.5	17.4	14.2	2.8	n.s.*
	Social relationships		438	14.9	3.0	107	59.5	19.1	13.5	3.1	4.5 E-05
	Environment		438	13.1	2.4	107	71.0	13.1	15.4	2.1	1.2 E-17
	Physical health	Non-healthy	470	13.1	2.7	107	69.1	14.0	15.1	2.2	7.0 E-12
	Psychological		470	12.5	2.6	107	63.5	17.4	14.2	2.8	6.8 E-09
	Social relationships		470	13.4	3.1	107	59.5	19.1	13.5	3.1	n.s.*
	Environment		470	12.8	2.4	107	71.0	13.1	15.4	2.1	5.8 E-23
Sweden											
	Physical health		–	–	–	119	70.3	18.0	15.3	2.9	–
	Psychological		–	–	–	119	63.7	17.3	14.2	2.8	–
	Social relationships		–	–	–	119	61.3	20.6	13.8	3.3	–
	Environment		–	–	–	119	72.5	14.0	15.6	2.2	–
United Kingdom											
	Physical health	Healthy	1324	76.5	16.2	42	64.1	20.1	14.3	3.2	1.5 E-06
	Psychological		1324	67.8	15.6	42	60.4	17.5	13.7	2.8	2.6 E-03
	Social relationships		1324	70.5	20.7	42	57.9	24.0	13.3	3.8	1.2 E-04
	Environment		1324	68.2	13.8	42	68.5	18.3	15.0	2.9	n.s.*
	Physical health	Non-healthy	1864	57.0	22.7	42	64.1	20.1	14.3	3.2	4.4 E-02
	Psychological		1864	58.3	19.3	42	60.4	17.5	13.7	2.8	n.s.*
	Social relationships		1864	62.8	23.2	42	57.9	24.0	13.3	3.8	n.s.*
	Environment		1864	64.9	16.9	42	68.5	18.3	15.0	2.9	n.s.*

Legend: n.s.* not significant after Bonferroni correction

(The values of the WHOQOL-BREF are shown with range 0–100 as well as range 4–20 for our study participants; reference population: value 0–100 for France, Germany and UK and value 4–20 for Netherlands and Poland)

### QOL per five diagnosis groups

QOL did not differ between the five diagnosis groups (TS female, KS male, CAH female, XY-DSD female, and XY-DSD male), in the domain *psychological health* [Table 4]. In *physical health* and *environment* men with KS reported lowest QOL (*p* < 0.001), in *social relationships* participants with XY-DSD identifying as males (*p* = 0.005). But all differences between highest and lowest scoring were smaller than half a standard deviation. The QOL of participants identifying other than male or female gender or not the typical gender one for the condition (*n* = 18) that had been excluded from the comparison of the diagnosis groups ranged from 12 to 92 on the 0 to 100 scale per domain; three of those scored lower than two standard deviation in one or two domains.

**Table 4:** WHOQOL-BREF per domain and diagnosis for those included into the regression analyses

WHOQOL-BREF domain	Turner Syndrome			Klinefelter Syndrome			CAH			XY-DSD female			XY-DSD male			*p*
Domain	n	Mean	SD	n	Mean	SD	n	Mean	SD	n	Mean	SD	n	Mean	SD	
	*301*			*213*			*221*			*194*			*93*			
Physical health																
Range 0–100	283	71.5	16.8	200	66.4	19.4	211	68.1	18.9	187	73.9	17.9	86	73.1	18.1	< 0.001
Range 4–20		15.4	2.7		14.6	3.1		14.9	3.0		15.8	2.9		15.7	2.9	
Psychological																
Range 0–100	284	63.7	16.1	201	63.3	17.8	211	65.6	18.4	188	64.6	18.9	87	63.2	20.2	0.663
Range 4–20		14.2	2.6		14.1	2.8		14.5	2.9		14.3	3.0		14.1	3.2	
Social relationships																
Range 0–100	283	65.9	18.6	201	59.4	21.9	211	64.8	20.5	188	62.6	20.7	87	57.2	23.4	< 0.001
Range 4–20		14.5	3.0		13.5	3.5		14.4	3.3		14.0	3.3		13.2	3.7	
Environment																
Range 0–100	283	73.9	12.9	201	69.9	14.9	211	74.1	15.7	187	75.0	15.4	87	72.2	13.8	0.005
Range 4–20		15.8	2.1		15.2	2.4		15.9	2.5		16.0	2.5		15.6	2.2	

(The values of the WHOQOL-BREF are shown with range 0–100 as well as range 4–20) (*n* = 1022)

### Variance in QOL

Our linear regression models explained 23% (for *social relationships*) to 45% (for *physical health*) of the variance of the QOL [Table 5]. The analyses showed that the individual’s health status is the most important predictor of QOL. A (very) good health status improved the QOL in all four domains significantly, the differences between very good health status compared to very bad health status ranged from 31.8 points in *social relationships*, [95% CI: 21.1, 42.5 points, *p* < 0.001] to 52.3 points in *physical health* [95% CI: 44.3, 60.2 points, *p* < 0.001]. Health status explained 62% to 81% (partial R^2^) of the R^2^ for each domain. Additional variance was explained by feelings about household income in all domains (partial R^2^: 10% for *social relationships* to 33% in *environment*), with living comfortable on present income being related to a better QOL. The relationship status with having a partner was positively associated with QOL in the domain *social relationships* (regression coefficient: 9.6 points [95% CI: 6.5, 12.6, *p* < 0.001]) with a partial R^2^ of 15%.

**Table 5:** Impact of health status and diagnosis group on QOL. Linear regression models of the four WHOQOL-BREF domains

		Physical health (*n* = 960)				Psychological (*n* = 963)				Social relationships (*n* = 962)				Environment (*n* = 961)			
Variable	Categories	ß	95% CI	*p*-value	p R^2^ (%)	ß	95% CI	*p*-value	p R^2^ (%)	ß	95% CI	*p*-value	p R^2^ (%)	ß	95% CI	*p*-value	p R^2^ (%)
Age		−0.1	−0.2, −0.0	0.006	5	−0.0	−0.1, 0.1	0.875	2	−0.2	−0.3, −0.1	0.006	5	0.0	−0.0, 0.1	0.360	1
Feeling about households income					14				14				10				33
Living comfortable on present income		0.0				0.0				0.0				0.0			
Coping with present income		−3.4	−5.3, − 1.5	< 0.001		−3.0	−5.2, − 0.9	0.006		−3.4	−6.0, − 0.8	0.009		−4.5	−6.2, −2.8	< 0.001	
(Very) difficult to live on present income		−9.0	− 11.8, −6.2	< 0.001		−6.9	− 10.0, − 3.8	< 0.001		− 5.2	−9.0, − 1.5	0.006		−12.2	−14.6, −9.7	< 0.001	
Relationship status					1				2				15				0
Being single		0.0				0.0				0.0				0.0			
Having a partner		0.3	−2.0, 2.5	0.806		2.9	0.4, 5.4	0.025		9.6	6.5, 12.6	< 0.001		−0.5	−2.5, 1.5	0.637	
Living with parents		− 0.5	−3.1, 2.2	0.727		1.2	−1.8, 4.2	0.425		3.2	−0.4, 6.7	0.081		−0.2	−2.5, 2.2	0.887	
Other		−1.6	−6.6, 3.5	0.544		1.1	−4.5, 6.7	0.697		−0.9	−7.6, 5.9	0.796		−2.0	−6.5, 2.4	0.366	
Health status					77				81				62				63
Very bad		0.0				0.0				0.0				0.0			
Bad		16.0	7.9, 24.2	< 0.001		7.9	−1.3, 17.0	0.092		5.8	−5.3, 16.7	0.305		14.1	6.9, 21.4	< 0.001	
Fair		28.4	20.7, 26.1	< 0.001		15.4	6.8, 24.0	< 0.001		11.6	1.3, 22.0	0.027		17.9	11.1, 24.6	< 0.001	
Good		41.7	34.0, 49.4	< 0.001		25.6	17.0, 34.2	< 0.001		21.9	11.6, 32.2	< 0.001		26.0	19.2, 32.8	< 0.001	
Very good		52.3	44.3, 60.2	< 0.001		38.3	29.4, 47.2	< 0.001		31.8	21.1, 42.5	< 0.001		34.1	27.1, 41.1	< 0.001	
Diagnosis					3				1				7				2
Turner		0.0				0.0				0.0				0.0			
Klinefelter		−0.7	−3.3; 1.9	0.593		1.8	−1.1, 4.7	0.221		−4.4	−7.9, −1-0	0.012		−1.8	−4.1, 0.5	0.124	
CAH		−3.2	−5.6; −0.7	0.012		1.7	−1.0, 4.5	0.219		−1.6	−4.9, 1.8	0.360		0.4	−1.8, 2.6	0.698	
XY-DSD female		1.1	−1.5; 3.7	0.398		−0.7	−3.6, 2.2	0.630		−4.8	−8.3, −1.4	0.006		0.2	−2.1, 2.4	0.893	
XY-DSD male		3.2	−0.2; 6.6	0.066		0.9	−2.9, 4.7	0.647		−7.9	−12.5, −3.4	< 0.001		−0.6	− 3.6, 2.4	0.678	
Model fit		R^2^ (%)		45		R^2^ (%)		28		R^2^ (%)		23		R^2^ (%)		33	

Diagnosis group explained very few additional variance (partial R^2^ < 8%) in all domains of QOL in the linear regression models. In the domain *physical health* a diagnosis of CAH in females explained some additional variance to health status [CAH regression coefficient: − 3.2 points, 95% CI: -5.6, − 0.7, *p* = 0.012] with slightly lower *physical health* compared to the other diagnosis groups. In the domain *social relationships,* a diagnosis of KS in males and a XY-DSD condition in females and males explained some additional variance to health status with a partial R^2^ of 7% with significantly lower QOL in social relationships compared to TS or CAH in females [KS male: regression coefficient: − 4.4 points, 95% CI: -7.9, − 1.0, *p* < 0.012; XY-DSD female: regression coefficient: − 4.8 points, 95% CI: -8.3, − 1.4, *p* < 0.006 and XY-DSD male: regression coefficient: − 7.9 points, 95% CI: -12.5, − 3.4, *p* < 0.001].

## Discussion

QOL life measures the adaptation of humans to all the circumstances that make life easy or burdensome. The measurement represents people’s individual perceptions about their position relative to other people and relative to their own expectations [3]. Negative life events generally show an impact on contemporary QOL whereas over time perceptions may show improved QOL even in the light of persisting negative circumstances, such as the diagnosis of a physical chronic health condition with a stable course. Life circumstances affecting several domains of life and causing daily acute stress such as pain [26, 27] or mental illness [28] generally affect QOL substantially and without a tendency of recovery.

The way that the WHO has conceptualized and developed measurements QOL taps a universal human understanding of a good life and is valid in various cultural contexts and physical environments [4]. Physical and mental health are a prerequisite to good QOL but do not account for it entirely. The term QOL is often used in a less precise way in medical literature but as an umbrella term for many psychosocial outcomes, often only relevant for a certain condition. In particular, instruments labeled health related quality of life (HRQOL) often contain measurements of functional capacities or health status. While these measurement are closer to the concept of a health impairment by attempting to assess the patients’ experiences as an essential part of the impact of the medical condition and it’s treatment, they underwrite the concept that only people with an objective good health status and role functioning have a good QOL [29]. In this study we went beyond the concept of HRQOL and measured QOL; health status and both physical and mental morbidity were considered as factors explaining parts of QOL as they tax people’s ability to cope and adapt, but not as integral and fixed part of QOL.

Given the fact that many studies pointed to impaired health outcomes in individuals with DSD, we were concerned about the fact that the complex issues affecting deeply rooted aspects of one’s personality such as gender identity, social relations and having a family, participation in society may result in overall low QOL.

This study is the largest report of individuals within the broad definition of DSD with five diagnosis groups including female TS, male KS (including male XYY), female CAH, female XY-DSD and male XY-DSD. The QOL indicators found that the groups were similar in psychological health, slightly poorer in physical health (not unexpected for individuals with chronic medical conditions), slightly better for environmental health, but with social relationships scoring lower than country reference populations. In *physical* and *psychological health* the differences to the healthy reference population are generally small and clinically not relevant [30]. In most cases, the scores are very different from the much lower scores of people with chronic physical or mental health conditions. Linear regression analysis indicated that health status was the most important predictor of QOL with variance being related to perceptions of income and relationship status. Diagnosis did not explain much of the variance in QOL.

The positive outcomes provide data consistent with an expectation of the potential for good QOL among those with these conditions. This further is consistent with the continuing goal of providing the best medical care for the underlying condition as well as any comorbidity to optimize overall health status and to enhance self-worth, and expectation of a good QOL [2].

However, in this study QOL is significantly reduced in the domain *social relationship* and in some countries even worse than for patients with other physical or mental chronic conditions. The questions of the *social relationships* domain include items very salient to the condition DSD: “How satisfied are you with your personal relationships?”, “How satisfied are you with your sex life?”, “How satisfied are you with the support you get from your friends?” [4, 5]. In this category we found a significant contribution of the fact whether or not the respondent had a partner, no matter whether they were married or living together. Only one third of the sample in this study (with 80% between the ages of 20 and 64 years) reported to have a partner. A large population based study from Britain has shown that, in addition to partnership issues, poor health contributes to decreased sexual activity and satisfaction; however, few seek clinical help [31]. The authors conclude that sexual lifestyle advice should be a component of holistic health care for patients with chronic ill health and this should be true in the DSD population as well [31]. Shame about the condition, concern about genital development, lack of enjoyable psychosexual experiences, lack of a family of one’s own and impaired participation in society may impact these results. The data analyzed do not provide information to assess broader family support nor personality characteristics. It could be anticipated that positive parental support since infancy and also having a personality that can adapt to adverse life events, including the underlying condition and therapy would be positive factors toward a good QOL. In further analysis we will explore all the factors potentially related to this finding.

QOL related to people’s living *environment* seems to carry no difference to the perceptions of healthy reference populations. The dsd-LIFE study sample included individuals with slightly higher education levels compared to the European Social Survey (ESS), especially in Sweden and the UK and slightly lower education levels in Poland [18]. The lower levels of QOL in the domain *social relationships* cannot be explained by overall worse living environments; availability of enough financial means does show an association with QOL in general but is not specific to this study sample.

Differences in QOL among the various subgroups were small and not clinically significant. Above and beyond the contemporary health status the specific diagnosis does not contribute much to explain differences in QOL. The findings point to the need to provide holistic and multidisciplinary care addressing all health problems, whether or not they are related to DSD or unrelated. As most tertiary care centers and clinics are focusing on specialty care they must establish efficient and safe networks of multidisciplinary teams and coordinate various systems of health care and social services. Given that these are rare health conditions, the individual may not have a medical home to address the complex health issues of the condition itself or squeal cause.

### Comparison to other studies

The study most comparable and relevant to our study was conducted in Brazil [14]. 56 adults with 46, XX DSD of whom seven identified as male and 88 with 46, XY DSD of whom 34 identified themselves as males took part in the single tertiary centre study. QOL was measured by the WHOQOL-BREF as in our study. The authors found no difference to the urban Brazilian general population, males scored even better in the domain psychological health compared to the general population. In the 46, XY DSD group males reported better QOL than females in all domains, but the difference failed to reach significance in the domain of social relationship. Interestingly, individuals that changed gender female-to-male pre- or postpubertal did not report any impairments in QOL [11]. Another study investigating QOL using the WHOQOL-BREF was conducted in China [13]. Included were 87 young women, aged 13 to 38 years, with TS, AIS, complete GD and CAH from a single gynecological centre. Compared to healthy Chinese urban population QOL was not reduced in any of the four domains and better than Chinese urban population with other chronic diseases in the domains of physical health, social relationship and environment [13]. Another study corresponds to our sub-sample of female 46, XY DSD conditions: 43 Caucasian adult females, aged 18 to 57 years (34 CAIS, four 5alpha-reductase and other diagnoses others) reported their QOL by using the WHOQOL-BREF [12]. The comparison group consisted of 43 females matched by age and education without any history of a medical health conditions. The study group reported a significant better QOL in physical health than controls. Similar to our findings QOL showed to trend to be lower in social relationships, but differences failed to reach significance. The authors did not find any differences in psychological health and environment between study and control group [12]. In Denmark the QOL of 70 women with DSD has been compared with controls matched on age, sex and school education by using the Quality of Life Assessment of Growth Hormone Deficiency in Adults (QOL-AGHDA), Danish version [10]. Nearly all of the participants had a diagnosis of CAH and only some 46,XY females with DSD conditions and varying degrees of virilisation. The authors report lower QOL in patients than in controls except for a small group of individuals with CAIS who reported much better QOL. Given that the QOL-AGHDA is an instrument developed for people with growth hormone treatment the finding is not surprising. Similarly the use of a disease specific questionnaire in healthy individuals might limit the interpretation of the findings [10]. Regarding those with sex chromosome DSD, Carel reported no differences in HRQOL, measured by using the SF-36, in 568 French young adult women with TS compared to the general female population [16]. But cardiac and otologic problems that affected one quarter of all participants were associated with lower scores in the SF-36 dimensions [16]. The German multicenter clinical evaluation study used the SF-36 in 110 adult participants, aged 17 to 62 years, with 46,XX and 46,XY DSD, but included all individuals identifying as females, males or other gender [15]. The study found similar scores compared to the German reference data in most domains and better HRQOL in the physical domain and slightly lower in mental HRQOL, but failed to reach statistical or clinical significance. The study reported no significant differences between the diagnostic subgroups with a trend to lowest scores in males with 46, XY DSD, failing to reach significance most likely due to small sample size [15].

The strength of dsd-LIFE is that by using a generic QOL measure, the WHOQOL-BREF, following the WHO definition of health, this is the first study ever allowing comparison to the general population and comparisons between diagnoses groups.

Limitations of this study, as noted above, include the lack of assessment of broad social support, including parental and other family support throughout life with the domain of *social relationships* containing only three items. Further, the Cronbach’s alpha and other psychometric properties are not as good as for the other three domains. Also, comparisons with the country-specific reference data must be interpreted with caution, taking into account the timeframe with some reference data being more than a decade older than our study data and the characteristics of the various samples [19–23]. The recruited sampling for reference of the WHOQOL-BREF appear to be representative for the general population only for France [20] and Germany [21], whereas healthy and non-healthy matched control groups for the best available reference for Poland and the Netherlands.

## Conclusion

Most people with DSD adapt well to their life circumstances and report a good QOL. Special focus should be on the deficits in the social relationships compared to the general population. It should be emphasized that diagnosis does not explain additional variance in QOL. But across all conditions, contemporary health status explains most of the variance in QOL, and often the health status is not directly related to the condition itself but may be classified as co-morbidity. This finding has major impact on the organization of care; individuals affected need both highly specializing care as well as a medical home comprehensively addressing all issues of health and collaborating with subspecialists.

## Abbreviations

AIS: Androgen insensitivity syndrome
CAH: Congenital adrenal hyperplasia
DSD: Disorders/differences of sex development
ESISCED: European survey version of ISCED (International Standard Classification of Education)
GD: Gonadal dysgenesis
HRQOL: Health related quality of life
KS: Klinefelter syndrome
PRO: Patient reported outcome
QOL: Quality of life
TS: Turner syndrome
UK: United Kingdom
WHOQOL-BREF: World Health Organisation Quality of Life – Short version
